# Novel insights into the roles and therapeutic implications of MUC1 oncoprotein via regulating proteins and non-coding RNAs in cancer

**DOI:** 10.7150/thno.63654

**Published:** 2022-01-01

**Authors:** Wei Li, Yineng Han, Chenmin Sun, Xue Li, Junhua Zheng, Jianping Che, Xudong Yao, Donald Kufe

**Affiliations:** 1 Department of Urology, Shanghai Tenth People's Hospital, Tongji University, Shanghai, China.; 2 Urologic Cancer Institute, Tongji University School of Medicine, Shanghai, China.; 3 Department of Orthodontics, Peking University School and Hospital of Stomatology, Beijing, China.; 4 Department of Anesthesiology, Tongren Hospital, Shanghai JiaoTong University School of Medicine, Shanghai, P. R. China.; 5 Department of Pathology, Beijing Chao-Yang Hospital, Capital Medical University, Beijing, China.; 6 Department of Urology, RenJi Hospital, Shanghai JiaoTong University School of Medicine, Shanghai, China.; 7 Dana-Farber Cancer Institute, Harvard Medical School, Boston, MA.

**Keywords:** MUC1, proteins, non-coding RNAs, clinical application

## Abstract

Mucin 1 (MUC1) is a heterodimeric transmembrane glycoprotein that protects epithelial cells in mammals. The transmembrane C-terminal subunit (MUC1-C) plays a crucial role in oncogenesis. As an oncoprotein, MUC1-C regulates a number of proteins that are associated with tumorigenesis by interacting with oncoproteins, transcription factors, coactivators, etc., inducing proliferation, epithelial-mesenchymal transition (EMT), invasion, stemness, immune evasion, and drug resistance. Moreover, MUC1-C modulates the expression of non-coding RNAs (ncRNAs), which further regulate carcinogenesis by directly binding to specific proteins. ncRNAs can also affect MUC1 protein expression by targeting the MUC1 mRNA 3′ untranslated region (UTR). A series of ncRNAs can modulate cancer development by regulating MUC1-C. This review focuses on the interaction of MUC1-C with proteins and ncRNAs in cancer progression. We also summarize the recent advances in immunotherapy with a focus on therapeutic approaches based on MUC1-C and nanocarrier complexes for cancer treatment.

## Introduction

Mucins are a family of high-molecular-weight glycoproteins with *O*-glycosidic linked oligosaccharides attached to serine or threonine residues of a core protein backbone. The human mucin family can be divided into secreted mucins (MUC2, MUC5AC, MUC5B, MUC6, MUC7, MUC8, and MUC19) and transmembrane mucins (MUC1, MUC3A, MUC3B, MUC4, MUC12, MUC13, MUC15, MUC16, MUC17, MUC20, and MUC21) 1. Secreted mucins contribute to a protective mucus gel that serves as a physical barrier at the apical epithelial cell membrane. Transmembrane mucins also contribute to this protective barrier, and certain members of this subclass, such as MUC1, can transduce inflammatory, proliferative, and remodeling signals to heal wounds and maintain epithelial cell homeostasis 2, 3.

**Figure 1:**
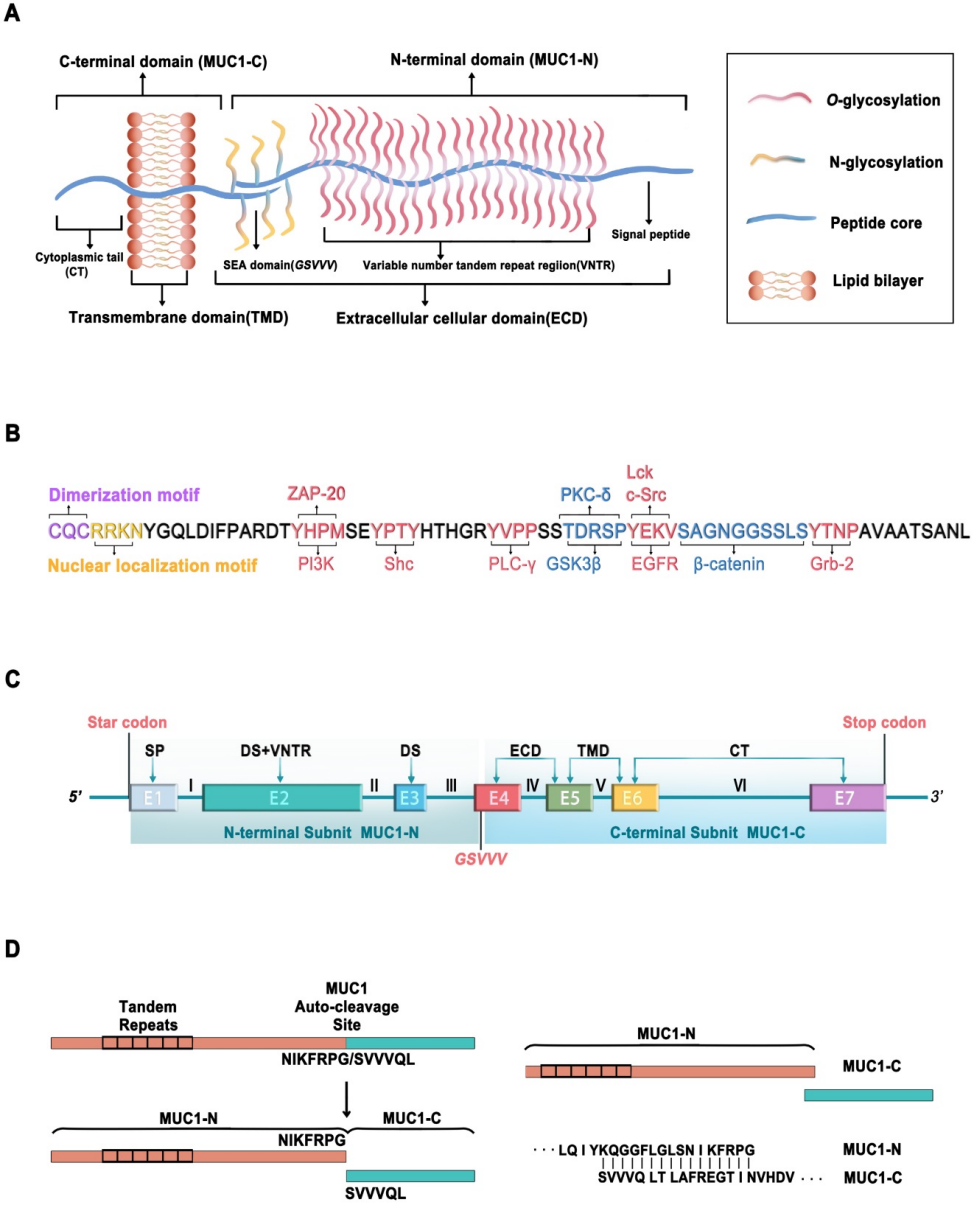
**Schematic representation of MUC1.** (A) Structure of MUC1, consisting of MUC1-C N-terminal (MUC1-N) and C-terminal (MUC1-C). (B) Amino acid sequence of cytoplasmic tail of MUC1-C (MUC1-CT) containing potential protein binding sites of various kinases (Red) and adapter proteins (Blue). The c-terminal end is the motif CQC that is essential for MUC1-C dimerization and interaction with other proteins. The motif RRK is responsible for nuclear translocation via Nup-62. (C) Schematic representation of the MUC1-C gene, which consists of 7 exons (E1~E7) and 6 introns (I~VI). MUC1-N is encoded by exons 1-3 and MUC1-C encoded by exons 4-7. Exon 1 (E1) encodes the signal peptide (SP), E2 encodes the N-terminal degenerate sequence (DS) and the VNTR. E3 encodes the C-terminal DS. E4, E5, E6, and E7 encode the extracellular domain (ECD), transmembrane domain (TMD), and cytoplasmic tail (CT). MUC1-C is translated as a single polypeptide which is autoproteolytically cleaved into MUC-N and MUC1-C after translation at the GSVVV motif (red). (D) Schematic diagram shows that the MUC1-C protein autocleavage at the GSVVV site into the MUC1-N and MUC1-C subunits (left) and the putative junction site of MUC1-N and MUC1-C (Right).

MUC1, the first structurally characterized mucin, has a single-pass type I transmembrane domain with an *O*-glycosylated polypeptide backbone that extends up to 200-500 nm from the cell surface (Figure 1A) 4-6. MUC1 is encoded by a gene located on chromosome 1q21, which contains seven exons 7. MUC1 is translated as a single polypeptide, which is autoproteolytically cleaved into two subunits at a GSVVV motif in the sea urchin sperm protein, enterokinase, and agrin (SEA) domain (Figure 1C). In turn, the extracellular N-terminal subunit (MUC1-N) and the transmembrane C-terminal subunit (MUC1-C) form a stable noncovalent heterodimeric complex at the cell membrane 8.

MUC1-N is encoded by exons 1-4 and contains variable number tandem repeats (VNTRs) of 20 amino acids, which are rich in proline, threonine, and serine (PTS) residues that are extensively *O*-glycosylated 2. MUC1-N is also modified by *N*-glycosylation in a region close to the cell membrane, which contributes to its secretion, localization, and folding. The fully glycosylated MUC1-N subunit extends above the glycocalyx to form a physical barrier that protects cells from damage caused by toxins, changes in pH, pathogens, reactive oxygen species (ROS), and other forms of stress associated with exposure to the external environment 9.

MUC1-C is encoded by exons 4-7, comprising a 58-amino acid extracellular cellular domain (ECD), a 28-amino acid transmembrane domain (TMD), and a 72-amino acid cytoplasmic domain (CD) 10 (Figure 1A, C). MUC1-C is highly conserved in mammals 10, 11. Unglycosylated MUC1-C has a molecular weight of 17 kDa, which increases with the extent of *N*-glycosylation of the MUC1-C ECD up to ~25 kDa (Figure 1B). The conserved MUC1-CD contains 7 tyrosine residues, 4 of which can be phosphorylated by kinases and initiate signal transduction cascades 12. MUC1-CD contains binding sites for phosphoinositide 3-kinase (PI3K) 13, zeta chain-associated protein kinase-70 (ZAP-70) 14, Src homology 2 domain containing (Shc) 15, phospholipase C-γ (PLC-γ) 12, protein kinase C-δ (PKC-δ) 16, glycogen synthase kinase 3β (GSK3β) 17, lymphocyte-specific protein tyrosine kinase (Lck) 14, epidermal growth factor receptor (EGFR) 14, β-catenin 18, and growth factor receptor-bound protein 2 (Grb2) 19 (Figure 1B).

MUC1-CD also contains the motif CQC, which is essential for MUC1-C dimerization and interaction with other proteins 20, 21. The cell-penetrating peptide GO-203 is extensively used to inhibit MUC1-C dimerization, which can suppress MUC1-C function by binding directly to the MUC1-C CQC motif and effectively blocking the reactivity of this site 22, 23.

MUC1-N (KQGGFLGLSNIKFRPG) binds to MUC1-C (SVVVQLTLAFREGTINVHDV) to form a stable heterodimer, resulting in the expression of the MUC1-N/MUC1-C non-covalent complex at the apical cell membrane (Figure 1D) 8. MUC1-N/MUC1-C is typically expressed in the glandular or luminal epithelial cells of the mammary gland, stomach, gastrointestinal tract, esophagus, pancreas, breast, uterus, lungs, prostate, and hematopoietic cells 24. However, it is not expressed in the skin epithelium or mesenchymal cells 25. Specifically, it is expressed on the apical borders of normal secretory epithelial cells as a heterodimeric complex 26. The MUC1-N/MUC1-C complex is disrupted by stress forces in the glycocalyx, leading to the shedding of MUC1-N in the mucous barrier and activation of MUC1-C for the intracellular transduction of signals to re-establish homeostasis 27. Proteases such as matrix metalloproteinase 14 (MMP14) and a disintegrin and metalloprotease 17 (ADAM17) can serve as MUC1 sheddases to accelerate the autocleavage of the MUC1-N/MUC1-C heterodimer 28, 29. Following the release of MUC1-N, the MUC1-C subunit can interact with the effectors of inflammatory, proliferative, and repair responses associated with wound healing. MUC1-C forms a complex with receptor tyrosine kinases (RTKs) at the cell membrane. In cancer cells, MUC1-C undergoes endocytic recycling into the cytoplasm and is transported to the mitochondrial outer membrane to attenuate the apoptotic response to oxidative and genotoxic stress 2, 30, 31. During tumorigenesis, MUC1-C is also overexpressed in the nucleus, where it interacts with transcription factors such as nuclear factor κ-light-chain-enhancer of activated B cells (NF-κB) p65, transcription factor 4 (TCF4), and signal transducer and activator of transcription 3 (STAT3) 2, 32-34. Under normal conditions, the MUC1 heterodimer is positioned at the apical borders of secretory epithelial cells. However, in the response of epithelial cells to stress accompanied by a loss of polarity, MUC1 can interact with cell surface molecules that otherwise localize at the basolateral borders 35. The redistribution of MUC1 on the cell surface initiates downstream signal transduction pathways, including the PI3K/AKT, mitogen-activated protein kinase (MAPK), and wingless type (WNT) pathways 32, indicating that MUC1 may play a crucial role in the transcriptional regulation of genes associated with tumorigenesis.

## Regulatory mechanisms of MUC1-C and associated proteins in cancer

**Figure 2:**
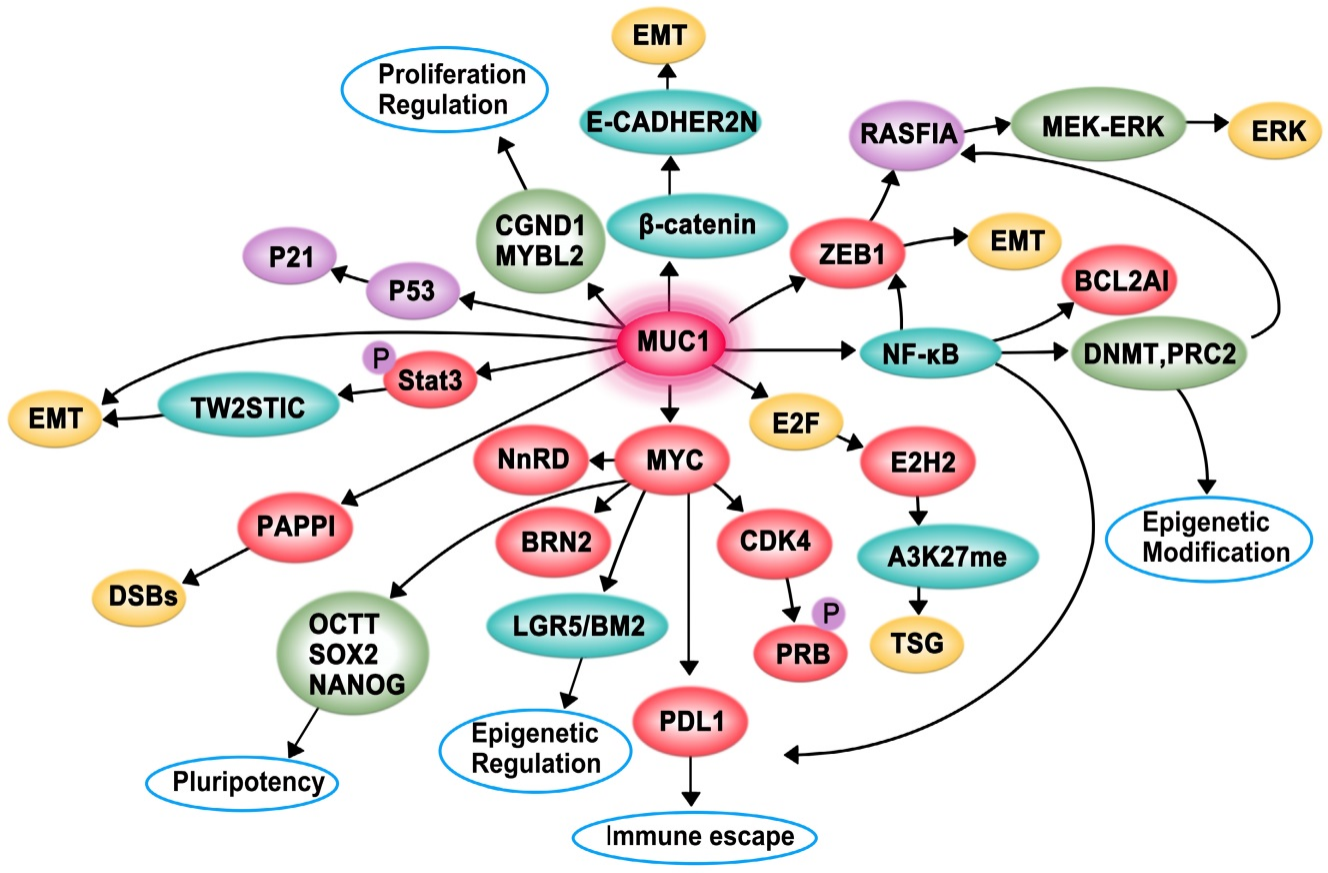
Proposed model of the function of MUC1-C in cancer cell proliferation, apoptosis, invasion, epithelial-mesenchymal transition (EMT), angiogenesis, immune evasion and drug resistance to promotes carcinogenesis

MUC1-C interacts with specific proteins to regulate tumorigenesis (Figure 2). It contributes to multiple hallmarks of cancer cells, including proliferation, apoptosis, invasion, epithelial-mesenchymal transition (EMT), angiogenesis, immune evasion, and drug resistance 36, 37.

## MUC1-C-mediated regulation of cancer cell proliferation and apoptosis

MUC1-C is directly associated with EGFR and translocates to the nucleus immediately after epidermal growth factor (EGF) stimulation. Subsequently, MUC1-C binds to cyclin D1 (CCND1) and v-myb myeloblastosis viral oncogene homolog-like 2 (MYBL2) promoters, contributing to the expression of G1/S phase genes 38. A recent study demonstrated that the increased expression of MUC1-C in androgen-dependent prostate cancer cells can inhibit androgen receptor (AR) axis signaling and induce the neural BRN2 transcription factor in prostate cancer by increasing MYC occupancy on the BRN2 promoter, which further promotes neuroendocrine prostate cancer 39. Moreover, MUC1-C forms a complex with zinc-finger E-box-binding homeobox 1 (ZEB1) on the RAS association domain family protein 1, isoform A (RASSF1A) promoter to recruit DNA methyltransferase (DNMT)-3b and suppress the transcription of RASSF1A, which is a tumor suppressor gene (TSG), further activating MEK→ERK signaling 40. Additionally, MUC1-C can promote colon cancer cell growth by downregulating TP53-inducible glycolysis and apoptosis regulator (TIGAR) expression in the AKT-mTOR-S6K1-eIF4A axis 41. A study found that patients with acute myeloid leukemia (AML) have an increased level of myeloid-derived suppressor cells (MDSCs) in their peripheral blood, and MUC1-C plays a critical role in driving extracellular vesicle (EV)-mediated MDSC proliferation 42.

The pro-apoptotic BAX protein containing a BH3 domain is essential for dimerization as well as the activation of the intrinsic apoptotic pathway. The cytoplasmic tail of MUC1-C (MUC1-CT) is associated with pro-apoptotic BAX in the response to genotoxic and oxidative stress. The MUC1-CT CQC motif binds directly to the BAX BH3 domain at Cys-62 and further blocks BAX dimerization, which activates the mitochondrial death pathway 43. The Hippo pathway is important for regulating proliferation, apoptosis, cell contact, and tissue repair. In transformed epithelial cells, MUC1-C can suppress the Hippo pathway and CRB3 expression by activating the oncogenic Yes-associated protein (YAP). Conversely, the interaction of MUC1-C with YAP induces the formation of YAP/β-catenin complexes and promotes the expression of the WNT target gene MYC 44.

## MUC1-C-mediated regulation of cancer cell invasion, EMT, and angiogenesis

Invasion is an early and necessary step of metastasis, which allows cancer cells to shed from the basement membrane and subsequently degrade the surrounding matrix and invade adjacent tissues and/or enter the bloodstream. EMT is a biological process in which cancer cells acquire invasive potential 45. MUC1-C can promote the invasiveness of breast cancer cells by modulating β-catenin localization and cytoskeletal dynamics 46. Moreover, MUC1-C can associate with platelet-derived growth factor (PDGF)-A, which drives tumor growth, angiogenesis, and metastasis in pancreatic ductal adenocarcinoma (PDA), contributing to the proliferation and invasion of pancreatic cancers in vivo and in vitro 47. The activation of STAT3 (pSTAT3) by MUC1-C induces the expression of TWIST1, which is an EMT transcription factor. MUC1-C directly binds to TWIST1 to form the MUC1-C/TWIST1 complex, which induces MUC1-C expression in an autoinductive loop 48. MUC1-C also forms nuclear complexes with poly(ADP-ribose) polymerase 1 (PARP1), which can catalyze the poly-ADP-ribosylation (PARylation) of itself and multiple target proteins to initiate the repair of single-strand and double-strand breaks in triple-negative breast cancer (TNBC) 23. Furthermore, MUC1-C can induce enhancer of zeste homolog 2 (EZH2) expression by activating the pRB→E2F pathway or the NF-κB pathway. Subsequently, H3K27 trimethylation (H3K27me3) is suppressed, and the activation of TSGs, such as BRCA1, is decreased 36, 49. MUC1-CT translocates to the nucleus to interact with β-catenin, thereby inhibiting the expression of E-cadherin and promoting the expression of EMT inducers such as Slug, Snail, Twist, and vimentin 50. MUC1-C can also induce EMT by activating the inflammatory NF-κB p65 pathway, which induces the transcription of ZEB1 and B-cell lymphoma 2-related protein A1 (BCL2A1) 37, 51. MUC1-C is involved in angiogenesis, and it can induce the expression of proangiogenic factors, including connective tissue growth factor (CTGF), PDGF-B, and vascular endothelial growth factor-A (VEGF-A), to promote endothelial cell tube formation and the synthesis of new blood vessels within the tumor. Overall, MUC1-C can promote cancer cell invasion, EMT, and angiogenesis, which synergistically contribute to cancer development 52.

## MUC1-C-mediated regulation of cancer cell immune evasion and drug resistance

The immune checkpoint protein programmed death ligand 1 (PD-L1) and MUC1-C are upregulated in TNBC, leading to aggressive pathogenesis. MUC1-C drives the transcription of PD-L1 by recruiting MYC and NF-κB p65 to the PD-L1 promoter, contributing to immune escape in TNBC. MUC1-C can also downregulate cluster of differentiation 8 (CD8), cluster of differentiation 69 (CD69), and granzyme B markers, which are associated with decreased survival 53. Moreover, MUC1-C plays a role in immune evasion by suppressing the p53 signaling pathway and CDKN1A/p21 54.

Tamoxifen is a common endocrine therapy for hormone receptor-positive breast cancer, and MUC1-C contributes to tamoxifen resistance through two mechanisms. On one hand, MUC1-C forms complexes with ERα on the estrogen-responsive RAB31 promoter and blocks the tamoxifen-induced decrease in ERα occupancy. On the other hand, MUC1-C recruits the coactivator CREB binding protein and activates the RAB31 promoter to upregulate RAB31 mRNA and protein levels 55. Moreover, MUC1-C attenuates cisplatin-induced mitochondrial apoptogenic factor release, caspase-3 activation, and apoptosis induction, thus conferring resistance to genotoxic anticancer agents 56.

## Regulatory mechanisms of MUC1-C and non-coding RNAs (ncRNAs) in cancer

ncRNAs, including microRNAs (miRNAs), long non-coding RNAs (lncRNAs), and circular RNAs (circRNAs), play vital roles in cancer 57. MUC1 can indirectly affect ncRNA expression by directly binding to specific proteins to regulate carcinogenesis (Table 1). In addition, accumulating evidence shows that the oncoprotein MUC1 is regulated by various ncRNAs in the modulation of cancer development.

## Regulation of microRNAs (miRNAs) by MUC1-C

MUC1-C can suppress the expression of miR-322, which has recognition sequences for the human galectin-3 3′ untranslated region (3′ UTR), thereby increasing the stability of galectin-3 mRNA 58. MUC1-C can also form a ZEB1/miR-200c regulatory loop and promote EMT and the invasion of breast cancer cells. Mechanistically, MUC1-C forms a complex with NF-κB p65, which occupies the promoter region of ZEB1 and induces the expression of ZEB1. Subsequently, MUC1-C directly binds to ZEB1, increasing the ZEB1 occupancy rate of the miR-200c promoter and leading to the suppression of miR-200c expression 59. Similarly, the direct interaction of MUC1-C with ZEB1, which interacts with the promoter of miR-200c/141, further reduces miR-200c/141 transcription 60. Moreover, MUC1-C can negatively regulate miR-34a and miR-200c expression, weakening their interference with PD-L1 translation and leading to the increased expression of PD-L1 in AML cells. MUC1-C can also block B lymphoma Mo-MLV insertion region 1 (BMI1) expression by binding to the highly conserved Z-boxes in the miR-200c promoter, thereby suppressing miR-200c expression, or binding directly to BMI1, which leads to the suppression of the CDKN2A/p16INK4a tumor suppressor 61. Mechanistically, MUC1-C does not affect miR-34a and miR-200c maturation but can alter the expression of DICER, which is an RNase III required for the generation of functionally competent miRNAs via c-Jun. In a previous study, miRNA array results demonstrated the upregulation of 340 functionally mature miRNAs in MUC1-silenced MOLM-14 cells, as well as the upregulation of 154 functionally mature miRNAs, including miR-34a and miR-200c, in MUC1-silenced human monocytic leukemia cells (THP-1) 62. MUC1-C promotes NF-κB p65 occupancy on the first intron of LIN28B and subsequently induces its transcription. Activated LIN28B suppresses the biogenesis of let-7 miRNAs, further inhibiting HMGA2 expression. The MUC1-C/LIN28B/let-7/HMGA2 axis promotes EMT traits and stemness in non-small cell lung cancer (NSCLC) 63. AR and MUC1-C have an inverse relationship in prostate cancer. AR signaling inhibits the expression of MUC1-C transcriptionally and post-transcriptionally. It can occupy the MUC1-C promoter and suppress the transcription of MUC1-C 64. In addition, AR can upregulate miR-125b, which has an effect on the MUC1-C 3′ UTR, further suppressing MUC1-C translation 64, 65. On the other hand, MUC1-C can downregulate AR mRNA via a post-transcriptional mechanism by upregulating miR-135b levels. MUC1-CT can also bind directly to AR in the DNA binding domain and form a complex that occupies the prostate-specific antigen promoter, inducing prostate cancer cell invasion and EMT 66. Mucin-type core 3 *O*-glycan induces p53 gene transcription by inhibiting the translocation of MUC1-C to the nucleus. Subsequently, p53 binds to the promoter region of miR-200c, finally leading to EMT in colon cancer cells 67. A study found that MUC1-C could drive the expression of c-myc mediated by miR34a in AML EVs to promote MDSC proliferation 42.

## Regulation of MUC1 by miRNAs

**Figure 3:**
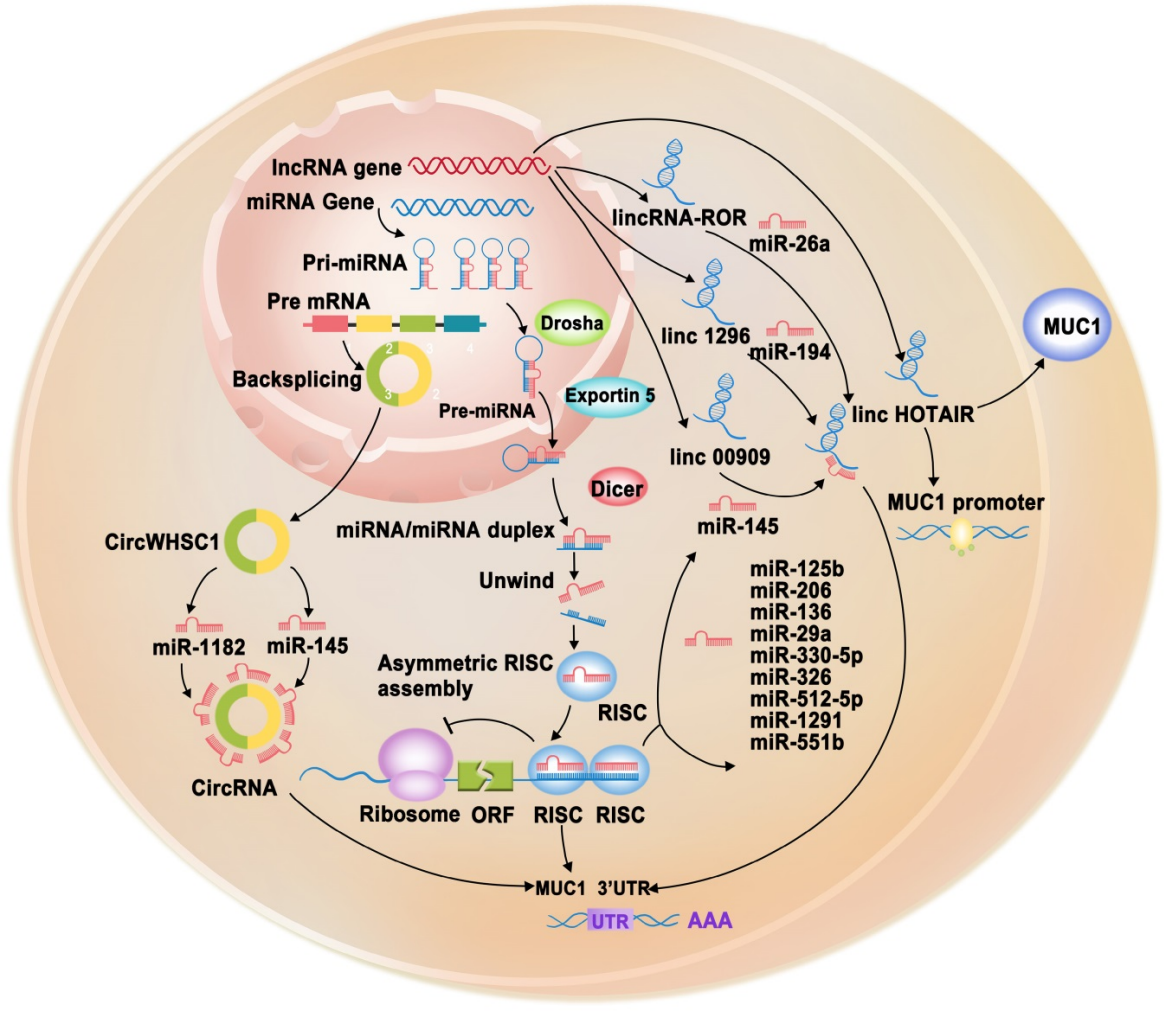
** Schematic representation of post-transcriptionally regulation of MUC1-C gene through non-coding RNAs (miRNAs, lncRNAs, circRNAs).** miRNAs (miR-125b, miR-145, miR-206, miR-136, miR-29a, miR-330-5p, miR-326, miR-512-5p, miR-1291, miR-551b) target the 3'UTR of MUC1-C gene to cause its degradation or translation repression. LncRNAs (LINC00909, LINC01296, lincRNA-ROR) serve as endogenous sponges of corresponding miRNAs to regulation MUC1-C gene expression. LncRNA HOTAIR can promote MUC1-C protein expression and promoter activity. circRNA circWHSC1 could sponge miR-145 and miR-1182 to increase MUC1-C expression, further promoting cancer progression.

miRNAs are a class of small ncRNAs approximately 22 nucleotides in length 68. By interacting with the 3′ UTR of mRNAs (through partial sequence homology), miRNAs mediate gene silencing via mRNA degradation or translation suppression, often serving as negative regulators of gene expression 69. Given their unique targeting capabilities, each miRNA can have more than 100 targets 70. miRNAs can suppress MUC1 protein expression but do not affect mRNA expression by targeting the 3′ UTR of MUC1 (Figure 3, Table 1). miR-125b is the first reported ncRNA that can post-transcriptionally regulate MUC1 expression. MUC1 contains the seed sequence of miR-125b in the 3′ UTR, and loss of miR-125b expression in breast cancer cells leads to the overexpression of MUC1 65. miR-145 also functions as a tumor suppressor by directly targeting the 3′ UTR of MUC1 to suppress its protein expression; however, it acts in a cell-specific manner. miR-145 can suppress ovarian cancer cell growth and invasion 71. In addition, miR-145 can inhibit the growth of the breast cancer cell lines MCF-7 and HCT-116; however, it does not affect metastatic breast cancer cell growth 72. Nevertheless, miR-145 can markedly suppress the invasion of these cells. Moreover, miR-125b and miR-145 do not affect MUC1 protein expression in H23 and H1793 lung adenocarcinoma cells 73. In gastric cancer, miR-206 can directly bind to the 3′ UTR of MUC1 and inhibit the expression of MUC1, thereby suppressing proliferation, migration, and invasion and inducing apoptosis 74. miR-136 can reduce the survival rate, suppress the colony formation ability, and induce the apoptosis of esophageal squamous cell carcinoma cells under irradiation by negatively regulating MUC1 75. In pancreatic cancer cell lines, miR-29a and miR-330-5p can inhibit cell proliferation, migration, and invasion and sensitize pancreatic cancer cells to gemcitabine by directly binding to the 3′ UTR of MUC1 to suppress its expression 76. miR-326 can suppress cell proliferation, colony formation, migration, and invasion, induce G0/G1 cell cycle arrest, and promote apoptosis in prostatic carcinoma cells by directly targeting the 3′ UTR of MUC1 77. miR-1226 can directly target the 3′ UTR of MUC1 mRNA and downregulate endogenous MUC1 protein levels in human breast cancer cell lines, which can increase ROS, lower the mitochondrial transmembrane potential, and ultimately decrease cell survival. These findings suggest that miR-1226 may act as a tumor suppressor by downregulating MUC1 expression to induce cell death 78. miR-512-5p has been found to downregulate MUC1 expression, further increasing the apoptosis and reducing the survival rate of cervical cancer cells 79. In esophageal cancer, miR-1291 can target the seed region of the 3′ UTR of MUC1 to suppress MUC1-C expression 80. MUC1 is also involved in acquired chemoresistance via the miR-551b/catalase/ROS axis. Here, MUC1 is regulated indirectly but not by miRNAs through 3′ UTR interactions. miR-551b can target the 3′ UTR of catalase mRNA and decrease catalase expression, which further increases cellular ROS accumulation. ROS post-transcriptionally upregulate MUC1 expression by suppressing lysosomal degradation, which activates the EGFR-mediated cell survival cascade involving AKT/c-FLIP/COX-2 and enhances chemoresistance 81.

## Regulation of MUC1 by lncRNAs

lncRNAs can function as a sponge for miRNAs to regulate various target genes, including MUC1, playing vital roles in various cellular processes (Figure 3, Table 1) 57. For example, LINC00909 could serve as an endogenous sponge of miR-194, thereby upregulating MUC1 expression, which further promotes the proliferation and invasion of glioma cells 82. *O*-glycosylation is essential for many biological processes. However, abnormal *O*-glycosylation promotes cancer cell invasion, metastasis, and recurrence 83. A study found that LINC01296 could function as a competitive endogenous RNA (ceRNA) that interacts with miR-26a, which negatively regulates *N*-acetylgalactosaminyltransferase 3 (GALNT3). GALNT3 can modulate colorectal cancer progression by catalyzing the *O*-glycosylation of MUC1-C 84. In TNBC cell lines, overexpressed lincRNA-ROR has been observed to act as a sponge for miR-145, subsequently upregulating MUC1-C expression. Increased MUC1 expression can inhibit the re-localization of β-catenin from the nucleus to the cytoplasm and reduce E-cadherin/β-catenin complex formation and E-cadherin membrane localization. The lincRNA-ROR/miR-145/MUC1/E-cadherin axis explains the invasion and metastasis mechanisms in TNBC cell lines 85. Polyphyllin I (PPI) is an active ingredient obtained from *Rhizoma Paridis* saponins, which exhibits biological activity against various types of cancer 86-88. PPI was recently found to inhibit castration-resistant prostate cancer growth by reducing p65 and MUC1 protein levels and downregulating lncRNA HOX transcript antisense RNA (HOTAIR) expression. Further investigation revealed that silencing HOTAIR could inhibit MUC1, whereas overexpression of HOTAIR could neutralize PPI-inhibited MUC1 protein expression and promoter activity 89.

## Regulation of MUC1 by circRNAs

circRNAs are a naturally occurring class of ncRNAs with a covalently closed continuous loop without 5′ caps and 3′ tails 90. circRNAs have been reported to play important roles in various diseases and function as potential biomarkers in cancer diagnosis. In particular, they have attracted increasing attention because they can function as miRNA sponges, gene transcription and expression regulators, and RNA-binding protein sponges 91. At present, there is limited information on the regulation of MUC1 by circRNAs. Only one study reported that circWHSC1 could sponge miR-145 and miR-1182 to increase MUC1 expression, further promoting ovarian cancer progression 92 (Figure 3, Table 1).

## Clinical applications of MUC1

### Immunotherapeutic approaches with MUC1-C as a target

Due to its potential as a cancer target, an increasing number of studies have focused on MUC1-C. One study designed a first-class monoclonal antibody (mAb), named 3D1, which could selectively bind to the surface of human cancer cells expressing MUC1-C with a high affinity 93. The results showed that mAb 3D1 and humanized mAb (humAb) 3D1 could be used as effective antibody-drug conjugates (ADCs) to deliver a payload, monomethyl auristatin E (MMAE), to cancer cells and tumors in mouse models. The mAb 3D1-MMAE ADC killed MUC1-C-positive cells in vitro, was non-toxic to MUC1-C transgenic mice, and was active against human HCC827 lung tumor xenografts. Therefore, humAb 3D1-MMAE ADC may be used as a treatment method for MUC1-C-overexpressing cancers 9. MUC1-C can promote PD-L1 transcription by recruiting MYC and NF-κB p65 to the promoter of PD-L1 in TNBC, leading to immune escape; thus, MUC1-C can be considered as a novel immunotherapeutic target for treating TNBC 53. GO-201 is a cell-penetrating peptide that contains the CQC motif and directly binds to MUC1-CT, subsequently blocking oligomerization. GO-203 is a second-generation configuration that is synthesized with all D-amino acids and is more stable in the presence of peptidases 22. As MUC1-C contributes to PARP1 activity, it is possible that targeting MUC1-C could influence olaparib sensitivity. An in vitro study demonstrated that GO-203 combined with olaparib could greatly enhance anti-tumor activity against TNBC 23. The combined use of GO-203 increased the sensitivity of AML cells to cytarabine (Ara-C) treatment via a survivin-dependent mechanism 94. One study used GO-203 to target an immunocompetent MUC1-C transgenic mouse, and the results demonstrated the suppression of PD-L1, upregulation of interferon-γ (IFN-γ), and enhanced effector function of CD8^+^ tumor-infiltrating lymphocytes 95. In addition, to reduce delivery frequency and prolong drug exposure, GO-203 was encapsulated in novel polymeric nanoparticles (GO-203/NPs). The results indicated that the effects of weekly administration of GO-203 nanoparticles to mice with allogeneic or xenograft tumors were comparable to those of daily administration of GO-203 96. A combination of GO-203 and the DNMT1 inhibitor decitabine can effectively decrease DNMT1 levels and the survival rate of AML cells considering that MUC1-C is an attractive target for the epigenetic reprogramming of AML cells, which can significantly reduce gene promoter-specific DNA methylation and further inhibit the expression of tumor suppressor genes, such as breast-cancer susceptibility gene 1 (BRCA1), cadherin 1 (CDH1), and phosphatase and tensin homolog deleted on chromosome 10 (PTEN) 97. Similarly, a combination of GO-203 and decitabine can increase ROS generation and decrease scavenger molecule levels, including nicotinamide adenine dinucleotide phosphate (NADP), reduced nicotinamide adenine dinucleotide phosphate (NADPH), glutathione, and TP53-induced glycolysis and apoptosis regulator (TIGAR), which are vital for intracellular redox homeostasis. This novel combination has been found to lead to marked gene-specific hypomethylation and the upregulation of ROS-generating enzymes, further increasing apoptotic death in cutaneous T-cell lymphoma (CTCL) 98. Moreover, MUC1-C could block the eIF4A cap-dependent translation of TIGAR, and GO-203 could induce the suppression of TIGAR by inhibiting the MUC1-AKT-mTOR-S6K1-eIF4A pathway, further inhibiting the growth of colon cancer cells 41. In TNBC cells, targeting MUC1-C with GO-203 can suppress the MEK→ERK and PI3K→AKT pathways by disrupting the stability of MCL-1, which is the main contributor to drug resistance in TNBC cells. A combination of GO-203 and ABT-737, which is a small molecule that targets BCL-2, BCL-XL, and BCL-w, may be a potential strategy for reversing MCL-1-mediated resistance in TNBC 99. These findings provide an experimental basis for further preclinical evaluation by targeting MUC1-C.

### Nanocarrier-based therapeutic approaches

**Figure 4:**
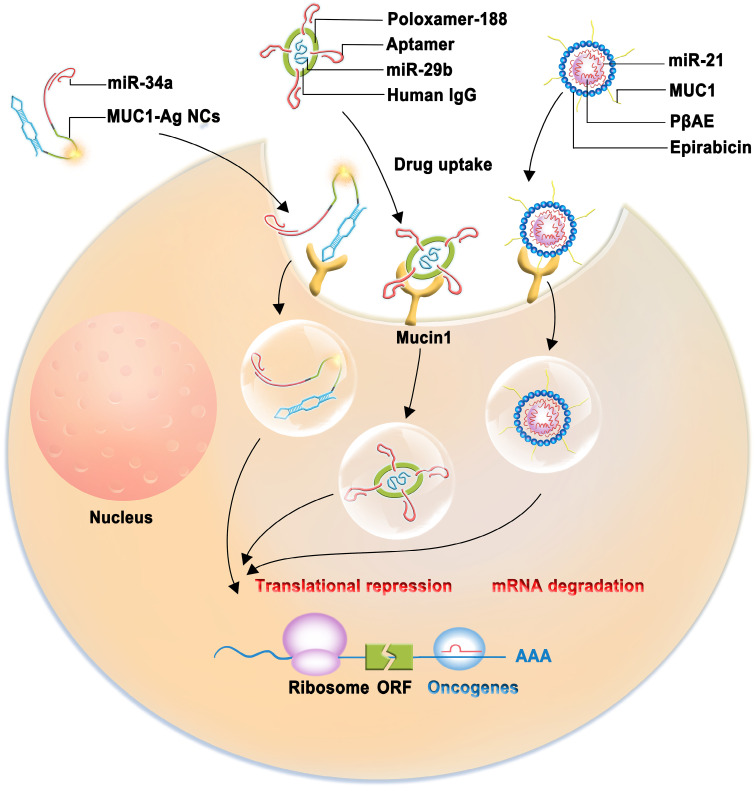
Schematic model of therapeutic approach which are combined with nanocarrier complex containing miRNAs (miR-34a, miR-21, miR-29b) with therapeutic effect and a tumor-targeting aptamer sequence specific to MUC1.

The importance of miRNAs as tumor suppressors is recognized in cancer therapy. However, tumor-suppressive miRNA replacements or mimics with less toxicity and fewer off-target effects are attracting increasing attention. Aptamers are single-stranded DNA (ssDNA) or RNA oligonucleotides isolated using the systematic evolution of ligands by exponential enrichment (SELEX) technique 100. Aptamers are efficient for delivering various therapeutic agents because of their small size, excellent biocompatibility, and ability to penetrate solid tumors without immunogenicity 101, 102. A novel multifunctional oligonucleotide nanocarrier complex, which consists of a tumor-targeting aptamer sequence specific to MUC1, a polycytosine region for fluorescent silver nanocluster (AgNC) synthesis, and a complementary sequence for miR-34a loading, has been designed (Figure 4). miR-34a is used because it can inhibit oncogene expression and induce cancer cell apoptosis. The intrinsic fluorescence of AgNC can be monitored to assess whether the constructed complex (MUC1-AgNCm-miR-34a) enters MCF-7 cells and whether this novel multifunctional AgNC-based nanocarrier improves the efficacy of breast cancer theranostics 103. MUC1-C aptamers mixed with poly(β-amino ester) (PβAE) can be highly responsive to pH changes, and the antimir-21 nanocomplex is less cytotoxic as it does not internalize into target and non-target cells 104. A study demonstrated that miRNA-29b and genistein loaded in MUC1 aptamer-functionalized hybrid nanoparticles (GMLHNs) could exhibit an anti-cancer effect on the NSCLC A549 cell line. The antiproliferative effect of GMLHNs is superior compared with that of individual genistein- and miRNA-29b-loaded nanoparticles, indicating that GMLHNs could be a potential treatment candidate for NSCLC 105-107. Moreover, a study combined MUC1-C aptamer and let-7i miRNA to establish a tumor tissue-specific delivery system to reverse paclitaxel-induced chemoresistance in OVCAR-3 ovarian cancer cells. The study demonstrated that the MUC1-C aptamer-miR-29b chimera (Chi-29b) could have an anti-tumor effect in xenograft tumor models and could play an anti-chemoresistant role by inhibiting cancer stem cell activation. Chi-29b was found to inhibit the growth of xenograft tumors by downregulating PTEN methylation, subsequent PTEN expression, as well as MAPK4 and IGF1 expression 108. Moreover, the Chi-29b chimera was found to exhibit an anti-tumor effect by inducing OVCAR-3 cell apoptosis 109.

## Perspectives

Recent studies have shed light on the MUC1-C regulatory network in carcinogenesis. Several ncRNAs have been reported to post-transcriptionally regulate MUC1-C by targeting its 3′ UTR, and in turn, MUC1-C regulates miRNAs to induce downstream cellular reactions. Moreover, the clinical effects of MUC1-C inhibitors alone or in combination with other anticancer drugs have been extensively studied to treat diseases. The potential of novel multifunctional oligonucleotide nanocarrier complexes composed of miRNAs that have been recognized as tumor suppressors for cancer therapy and a tumor-targeting aptamer sequence specific to MUC1-C has been widely investigated to minimize toxicity and reduce off-target effects. This therapeutic approach is expected to address the existing problems of current anti-cancer drugs in the near future. Overall, the identification of MUC1-dependent pathways is of potential importance in cancer research. This will pave the way for the development of targeted treatments with the aim of overcoming drug resistance and off-target effects, ultimately reducing the severity and progression of cancer.

## Figures and Tables

**Table 1:** Regulation mechanisms of MUC1-C and non-coding RNAs in cancer.

Non-coding RNA	Cancer	Function	Reference
miR-125b	Breast cancer	Promote DNA damage-induced apoptosis.	65
miR-145	Ovarian cancer	Growth and invasion	71
	Breast cancer	MCF-7 and HCT-116 cells growth	72
		Suppresses metastatic breast cancer cell lines invasion	73
miR-206	Gastric Cancer	Inhibits Proliferation, Migration, and Invasion	74
miR-136	Esophageal squamous cell carcinoma	Reduced the survival rate, suppressed colony formation ability and induced apoptosis	75
miR-29a, miR-330-5p	Pancreatic cancer	Inhibit cell proliferation, cell migration, cell invasion and sensitize pancreatic cancer cells to gemcitabine	76
miR-326	Prostatic carcinoma	Suppress cell proliferation, colony formation, migration and invasion, induced G0/G1 cell cycle arrest, and promoted apoptosis	77
miR-1226	Breast cancer	An increase in reactive oxygen species, ii) loss of the mitochondrial transmembrane potential, and iii) a decrease in cell survival.	78
miR-512-5p	Cervical cancer cells.	Increased apoptosis and reduced cell survival rate	79
miR-1291	Esophageal Squamous Cell Carcinoma	Cell Proliferation and Invasion to Promote Cell Apoptosis	80
miR-551b	Apoptosis resistance and chemoresistance	miR551b/catalase/ROS/MUC1-C to acquired apoptosis resistance and chemoresistance	81
LINC00909	Glioma cells	Sponge miR-194, promoting the proliferation and invasion	82
LINC01296	Colorectal cancer	Sponge miR-26a, negatively regulated GALNT3. GALNT3 catalyzed the *O*-glycosylated MUC1	84
lincRNA-ROR	Triple-negative breast cancer	Sponge miR-145, promote invasion and metastasis	85
LINC HOTAIR	Castration-resistant prostate cancer	Growth	89
circWHSC1	Ovarian cancer	Proliferation, migration and invasion, and inhibited cell apoptosis	92

## Abbreviations

ADCs: antibody-drug conjugates
ADAM17: a disintegrin and metalloprotease 17
AgNC: silver nanocluster
AML: acute myeloid leukemia
Ara-C: cytarabine
AR: androgen receptor
ASC: apoptosis-associated speck-like protein containing a CARD
BCL2A1: B-cell lymphoma 2-related protein A1
BMI1: B-lymphoma Mo-MLV insertion region 1
CCND1: cyclin D1
CD: cluster of differentiation
circRNAs: circular RNAs
COPD: chronic obstructive pulmonary disease
CSCs: cancer stem cells
CT: cytoplasmic tail
CTCL: cutaneous T-cell lymphoma
CTGF: connective tissue growth factor
DNMT: DNA methyltransferase
DSBs: double-strand breaks
ECD: extracellular cellulardomain
EGF: epidermal growth factor
EGFR: epidermal growth factor receptor
EMT: epithelial-mesenchymal transition
EMT-TF: epithelial-mesenchymal transition transcription factor
EV: extracellular vesicle
EZH2: enhancer of zeste homolog 2
GALNT: N-acetylgalactosamine transferases
GMLHNs: genistein loaded in MUC 1-aptamer-functionalized hybrid nanoparticles
Grb2: growth factor receptor-bound protein 2
GSDMD: Gasdermin D
GSK3β: glycogen synthase kinase 3β
H3K27me3: H3K27 trimethylation
HOTAIR: HOX transcript antisense RNA
humAb: humanized mAb
IL: Interleukin
IFN: interferon
IFN-β: interferon - β
IFN-γ: interferon-γ
Lck: lymphocyte-specific protein tyrosine kinase
lncRNAs: long non-coding RNAs
mAb: monoclonal antibody
MAPK: mitogen-activated protein kinase
MDSCs: myeloid-derived suppressor cells
MMAE: monomethyl auristatin E
MMP-14: Matrix metalloproteinases 14
miRNAs: micro RNAs
MUC1: Mucin 1
MYBL2: v-myb myeloblastosis viral oncogene homolog-like 2
MyD88: myeloid differentiation primary response gene 88
NADP: nicotinamide adenine dinucleotide phosphate
NADPH: nicotinamide adenine dinucleotide phosphate
NF-κB: nuclear factor κ-light-chain-enhancer of activated B cells
NLRP3: NOD like protein 3
NSCLC: non-small cell lung cancer
PARP1: poly-ADP-ribose polymerase 1
PARylation: poly-ADP-ribosylation
PDA: pancreatic ductal adenocarcinoma
PD-L1: programmed cell death-ligand 1
PI3K: phosphoinositide 3-kinase
PKC-δ: protein kinase C-δ
PLC-γ: phospholipase C-γ
PPI: Polyphyllin I
PRC1: polycomb represseses complex 1
PTS: proline, threonine, and serine-rich
PβAE: poly β amino ester
RASSF1A: Ras association domain family protein1 isoform A
RBP: RNA-binding proteins
ROS: reactive oxygen species
RTKs: receptor tyrosine kinases
SEA: sea urchin sperm protein enterokinase and agrin
SELEX: systematic evolution of ligands through exponential enrichment technique
Shc: Src homology 2 domain containing protein
ssDNA: single-stranded DNA
STAT3: signal transducer and activator of transcription 3
TCF4: transcription factor 4
TIGAR: TP53-inducible glycolysis and apoptosis regulator
THP-1: human monocytic-leukemia cells
TILs: tumor-infiltrating lymphocytes
TLR: Toll-like receptor
TMD: transmembrane domain
TNBC: triple-negative breast cancer
TSG: tumor suppressor gene
UTR: untranslated region
VEGF-A: vascular endothelial growth factor-A
VNTRs: variable number tandem repeats
WNT: wingless type
YAP: Yes-associated protein
ZAP-70: zeta chain-associated protein kinase-70
ZEB1: zinc-finger E-box-binding 1
